# Post-Vaccination Pharyngeal-Cervical-Brachial Variant of Guillain-Barré Syndrome

**DOI:** 10.7759/cureus.9804

**Published:** 2020-08-17

**Authors:** Funmilola T Taiwo, Daniel E Ezuduemoih, Philip B Adebayo

**Affiliations:** 1 Neurology, University College Hospital, Ibadan, NGA; 2 Internal Medicine, Lagos University Teaching Hospital, Lagos, NGA; 3 Neurology, Aga Khan University, Dar es Salaam, TZA

**Keywords:** pharyngeal-cervical-variant, guillain-barré syndrome, vaccination, mmr, case report

## Abstract

The pharyngeal-cervical-brachial (PCB) variant of Guillain-Barré syndrome (GBS) is very rare. It is characterized by weakness of the upper extremities associated with bulbar symptoms and facial diplegia. Documented cases were post-infectious, a post-vaccination occurrence has not been documented in the available literature. Even rarer is the occurrence of any variant of GBS following the mumps measles rubella (MMR) vaccine. The neurophysiological hallmark of PCB variant of GBS is a combination of myelinopathy and axonopathy, hence, its consideration as a subtype of the acute motor axonal neuropathy (AMAN) variant. It should be suspected in any case of acute-onset flaccid symmetrical weakness of the upper extremities, as early diagnosis and treatment are key to preventing fatal bulbar weakness. Here we report a case of a middle-aged man, who presented with features of PCB a fortnight after being vaccinated for MMR.

## Introduction

Guillain-Barré syndrome (GBS) is an acute, immune-mediated polyradiculopathy that is characterized by an acute-onset symmetrical flaccid muscle weakness with decreased or absent deep tendon reflexes. Pharyngeal-cervical-brachial (PCB) variant of GBS is a localized variant that presents with facial palsy, dysarthria, or dysphagia in addition to the weakness of the upper extremity and areflexia of the upper limbs [1]. These features should not occur in the settings of ophthalmoplegia, ataxia, altered consciousness, and prominent lower limb weakness. The PCB variant of GBS is a rare variant of the disease whose pathological hallmark is both demyelinating and axonal neuropathy [2].

Although two-thirds of cases of GBS are associated with antecedent infections, most of which are sporadic, other precipitants include surgery, pregnancy, cancer, and vaccinations [3]. However, post-vaccination GBS is not very common. Cases of localized variants of GBS following vaccination are even rarer. We report a case of PCB variant of GBS in a native African following mumps measles rubella (MMR) vaccination.

## Case presentation

A 55-year-old man presented with a three-day history of weakness of both upper extremities, which was noticed initially as difficulty in handling cutlery, which then progressed to difficulty with lifting both upper limbs. He also complained of a worsening moderate to severe pain around the neck and shoulders. The symptoms prompted his presentation to the ER, from where he was referred to the neurology outpatient department. A week before, he had complained of a fleeting tingling sensation in the feet which had resolved spontaneously. He had no prior fever, sore throat, diarrhea, surgery, or any other recent travel. He did not consume canned food, not an IV drug user, and drank alcohol on social occasions. Two weeks prior, he had received a vaccination for MMR. He had no underlying diabetes but took amlodipine for his hypertension.

His initial evaluation revealed symmetric flaccid paresis of both upper limbs with a total loss of handgrip bilaterally. His proximal muscle strength in the upper extremities was Medical Research Council (MRC) grade 4, and across all muscle groups in the lower legs was MRC grade 4. The neck flexors were weaker than the extensors at grade 3- and grade 3+, respectively. There was no muscle atrophy. The gag reflex was absent but there was no swallowing difficulty. There was no evident weakness of the respiratory muscles. He had no objective sensory findings. His peak flow rate was normal.

His cerebrospinal fluid (CSF) analysis done the following day showed no cytoalbumin dissociation (Table 1).

**Table 1:** Routine CSF analysis. CSF, cerebrospinal fluid

Parameters	Values	Lab reference
Appearance	Clear colorless	Clear colorless
Supernatant	Clear	Clear
White cell count (mm3)	Nil	0-5
Red cell count (mm3)	Nil	Nil
Protein (mg/dL)	38.13	15-45
Sugar (mmol/L)	3.16	2.22-3.89
Gram stain	No organism	Nil

**Figure 1:**
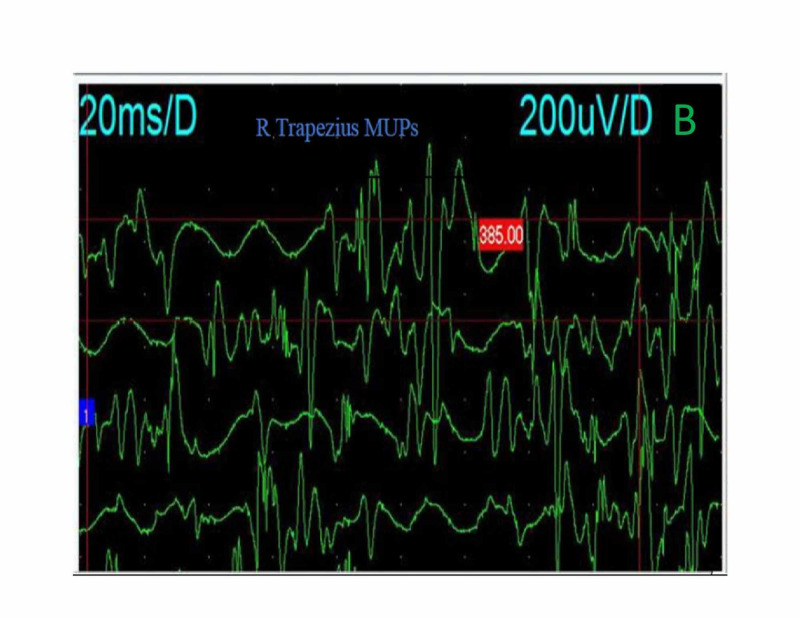
Needle EMG of the right trapezius showing neurogenic MUPs and increased polyphasia. EMG, electromyogram; MUPs, motor unit potentials

**Figure 2:**
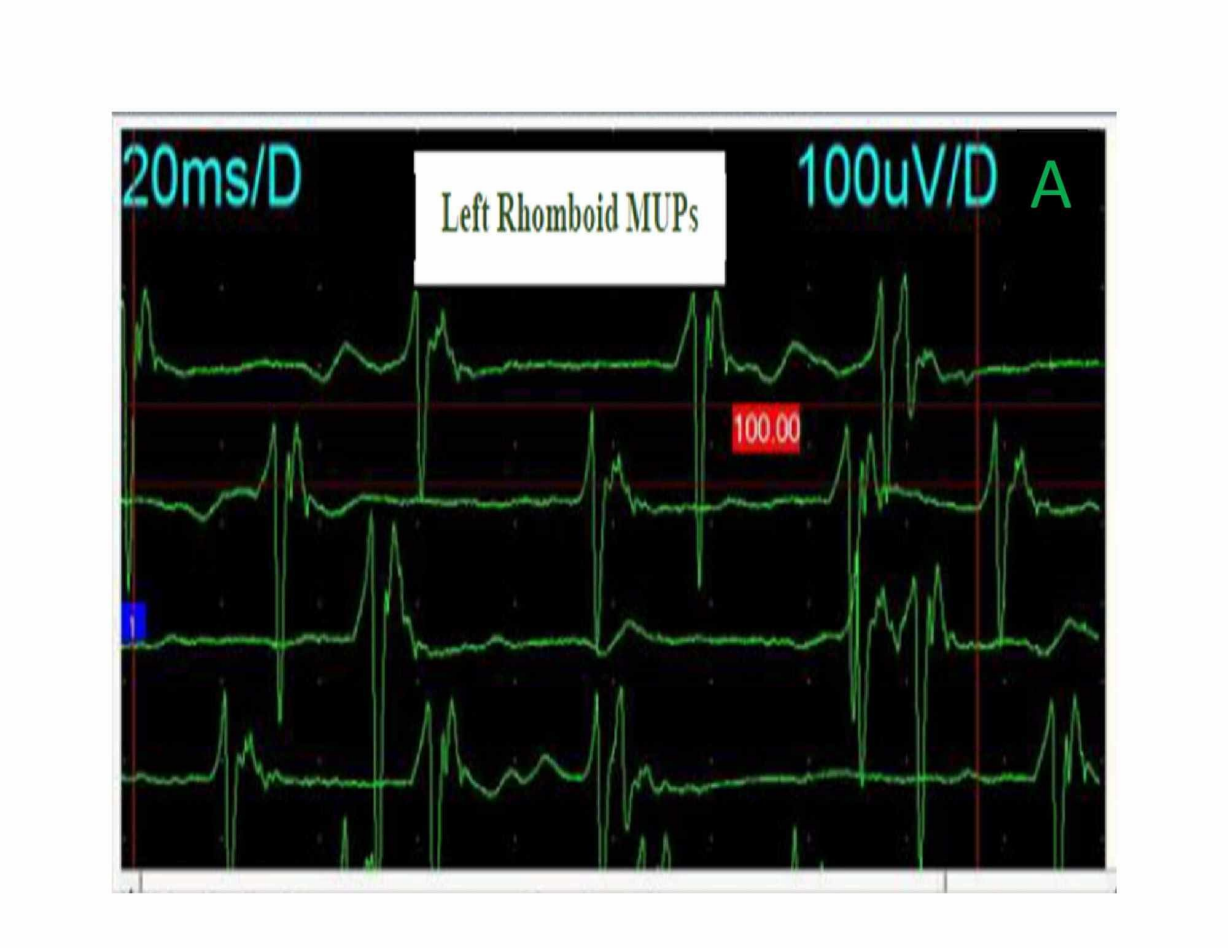
Needle EMG of the left rhomboid muscles showing neurogenic MUPs. EMG, electromyogram; MUPs, motor unit potentials

His first electrodiagnostic study showed conduction block of the right ulnar and left median compound motor action potential (CMAP) as can be seen in Table 2 and delay of the left median and both ulnar distal motor latencies (profound on the right). The median and ulnar F waves were absent. The right and left spinal accessory CMAPs amplitudes were decreased. Other blood works were within normal limits. He was commenced on intravenous immunoglobulin (IVIG) at 40 g daily for three days. Furthermore, we started carbamazepine at 200 mg twice daily in addition to oral tramadol (50 mg twice daily) and diclofenac (50 mg twice daily) to achieve adequate pain control. The patient commenced physiotherapy. A few days after admission, he developed diplopia, facial diplegia, and dysarthria, all of which resolved within a fortnight of onset. However, the proximal muscles of the upper limbs took a while to recover fully. After six weeks, his electrodiagnostic study was repeated, and it showed average values in all the nerves. The conduction block of the ulnar nerve had resolved (reversible conduction block) except for both spinal accessory nerves (Table 2). Needle electromyogram (EMG) that was performed on day 90 revealed neurogenic motor unit potential (MUP) with reduced activation and weak recruitment pattern of the right trapezius muscle and the left rhomboid muscles (Figures 1-2).

**Table 2:** Serial nerve conduction parameters of the patient. NP, not performed; CMAP, compound motor action potential; DML, distal motor latency; CV, conduction velocity; SNAP, sensory nerve action potential

Nerve conduction parameters	Day 3	Day 45	Day 90
Right median CMAP amplitude (mV)-Distal (Proximal)	9.65 (9.05)	10.87 (9.96)	NP
Left median CMAP amplitude (mV)-Distal (Proximal)	9.30 (3.80)	9.39 (7.96)	NP
Right median DML (ms)	3.10	3.18	NP
Left median DML (ms)	4.22	3.26	NP
Right median motor CV (m/s)	39.22	63.64	NP
Left median motor CV (m/s)	75.90	62.50	NP
Right ulnar CMAP amplitude (mV)-Distal (Proximal)	7.65 (--)	3.35 (3.22)	NP
Left ulnar CMAP amplitude (mV)-Distal (Proximal)	2.07 (0.41)	3.42 (3.04)	NP
Right ulnar DML (ms)	15.16	2.92	NP
Left ulnar DML (ms)	4.22	3.10	NP
Right ulnar motor CV (m/s)	--	32.24	NP
Left ulnar motor CV (m/s)	39.86	32.74	NP
Right spinal accessory amplitude (mV)	2.66	0.11	6.92
Left spinal accessory amplitude (mV)	2.54	0.03	1.72
Right median SNAP onset latency (ms)	2.70	2.39	NP
Left median SNAP onset latency (ms)	2.90	2.30	NP
Right ulnar SNAP onset latency (ms)	2.48	2.30	NP
Left ulnar SNAP onset latency (ms)	2.54	2.06	NP
Right median F wave (min V)	-	28.26	NP
Left median F wave (min V)	-	28.70	NP
Right ulnar F wave (min V)	-	32.24	NP
Left ulnar F wave (min V)	-	32.74	NP

**Figure 3:**
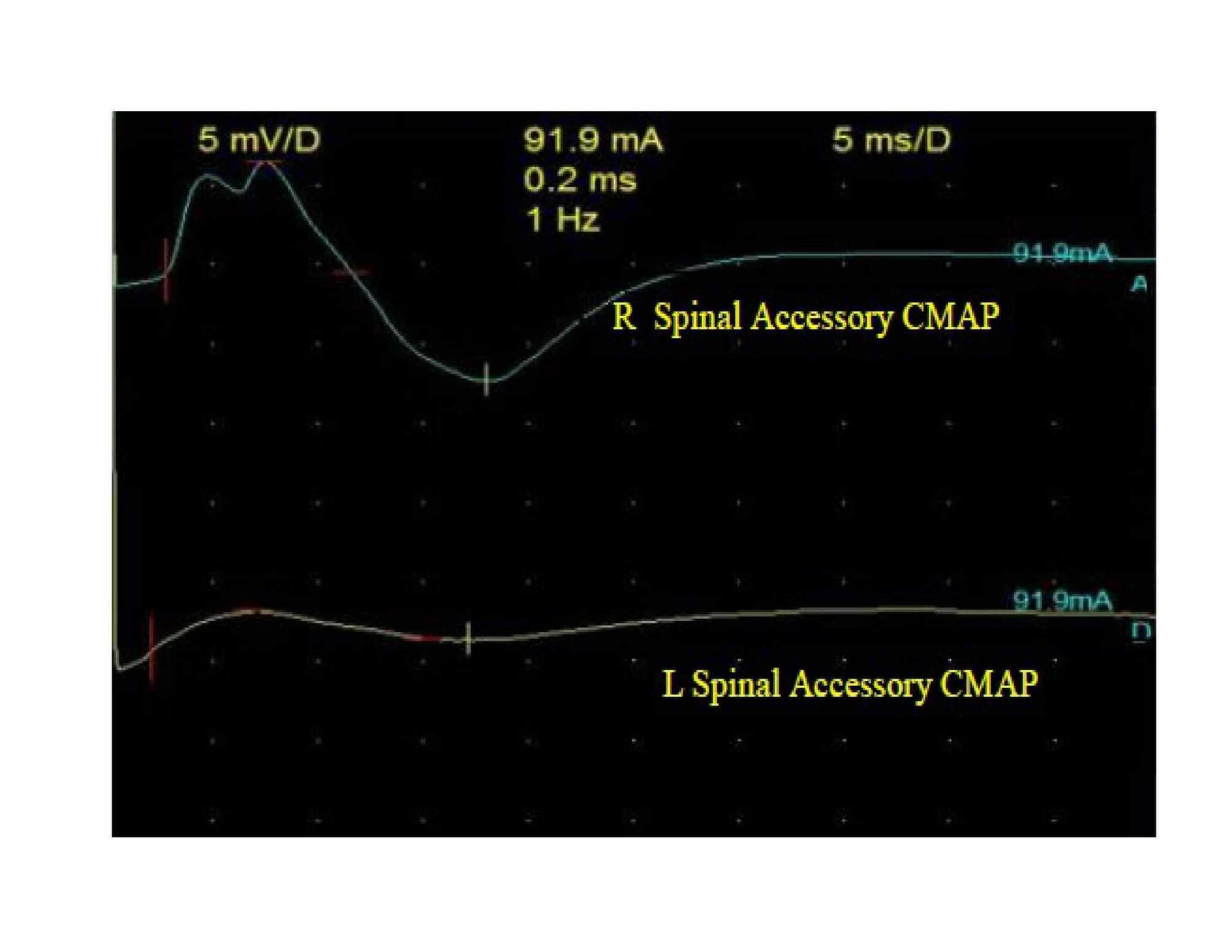
Right and left spinal accessory CMAPs at day 90. CMAP, compound motor action potential

At 90 days after onset, his right accessory nerve had recovered, but the left accessory nerve recovery lagged, showing temporal dispersion and decreased amplitude (Figure 3).

The patient eventually made a progressive full recovery over the following months. His neurological evaluation at six months revealed no deficit.

## Discussion

Patients with the PCB variant of GBS typically present with rapidly progressive oropharyngeal and cervicobrachial weakness associated with areflexia in the upper limbs and the presence of monospecific IgG anti-GT1a antibodies in the serum is typical [4]. However, the association with GM1b and GD1a has also been described. The electrophysiological hallmark of PCB is axonal conduction failure, and it is regarded as a continuation of acute motor axonal neuropathy (AMAN) variant of GBS [2, 5]. The clinical and electrophysiological profile of our patient was typical of PCB although we did not test anti-ganglioside antibodies as it is not required for diagnosis. The presence of diplopia in our patient suggested an ophthalmoparesis which has been found in about 40% of cases [4]. This variant of GBS is rare [6]. But rarer still is the association with vaccination. Hall et al. reported a case of PCB in a middle-aged man who had Vibrio cholerae vaccination but reports of post-vaccination PCB variant of GBS are scarce globally [1]. We are not aware of similar cases in sub-Saharan Africa.

Most cases of PCB variant of GBS in the literature are post-infectious. Nagashima et al. found a preceding infection in all the 100 patients with PCB in their study [4]. Seventy-one percent had a prior respiratory tract infection, and 30% had preceding diarrhea. Of this cohort, *Campylobacter jejuni* was the most common antecedent infectious agent (30%) followed by Cytomegalovirus (6%), while Epstein-Barr virus, *Mycoplasma pneumoniae*, and Hemophilus influenza accounted for 7%. Prior dengue fever has also been reported as a precursor of PCB [7].

Influenza vaccine is the most common antecedent vaccine that accounted for most post-vaccination GBS. The incidence of GBS rose by more than nine-fold during the 1976 flu vaccination season with the influenza A/H1N1, although no significant increase in GBS cases been witnessed since then [3]. MMR vaccine, hepatitis B vaccine, polio vaccine and diphtheria, tetanus, and pertussis (DTP) vaccines have been implicated as causes of post-vaccination GBS even though a direct linkage of GBS with MMR vaccine was not found in a Korean population [8]. The causal relationship (development of symptoms within six weeks of receiving the vaccine) and the absence of preceding alternative events make ours a particular case of post-vaccination PCB according to the WHO adverse events following immunization (AEFI) criteria [9].

The clinical course of PCB should be acute and monophasic [2]. Our patient’s disease evolved rapidly over days and plateau at two weeks. His bulbar and facial symptoms ultimately improved over 14 days following IVIG administration, albeit, his shoulder weakness took a while before total recovery. We attributed this to the axonal injury in the spinal accessory nerve evidenced by the neurogenic MUPs in the shoulder muscles. Pain is another common feature of GBS; as in our patient, a deep aching pain may be present in weakened muscles that patients could liken to sore, over-exercised muscles [4]. Dysesthesia pain in the neck and shoulders, which worsens over days, could reflect sensory nerve fiber involvement.

## Conclusions

Pharyngeal-cervical-brachial variant of GBS is a rare variant of the disease but its precipitation by vaccination is rarer. While the response to IVIG is typical for post-infections cases, it is unclear if the time to nadir and the response to IVIG are similar in post-vaccination cases. Furthermore, this variant of GBS should be remembered and ruled out, in patients who present with oropharyngeal and cervicobrachial weakness. Electrophysiology and CSF analysis are useful in diagnosis, but initial normal values do not rule out the disease. Clinical diagnosis is paramount.

## References

[1] Hall JN, Sheikh MN, Shumak SL (2014). Pharyngeal-Cervical-Brachial Variant of Guillain-Barre Syndrome. J Neurol Res.

[2] Wakerley BR, Yuki N (2014). Pharyngeal-cervical-brachial variant of Guillain-Barré syndrome. J Neurol Neurosurg Psychiatry.

[3] Donofrio P (2017). Guillan Barre Syndrome. Contin Lifelong Learn Neurol.

[4] Nagashima T, Koga M, Odaka M, Hirata K, Yuki N (2007). Continuous spectrum of pharyngeal-cervical-brachial variant of Guillain-Barré syndrome. Arch Neurol.

[5] Arai M, Susuki K, Koga M (2003). Axonal pharyngeal-cervical-brachial variant of Guillain-Barré syndrome without anti-GT1a IgG antibody. Muscle and Nerve.

[6] Ropper AH (1986). Unusual Clinical Variants and Signs in Guillain-Barré Syndrome. Arch Neurol.

[7] Pandey R, Jain R, Hussain S (2019). Pharyngeal-cervical-brachial variant of Guillain-Barré syndrome following dengue infection: A rare syndrome with rare association. Ann Indian Acad Neurol.

[8] Park YS, Lee KJ, Kim SW, Kim KM, Suh BC (2017). Clinical features of post-vaccination Guillain-Barré syndrome (GBS) in Korea. J Korean Med Sci.

[9] BCCDC. Communicable Disease Control Manual Chapter 2: Immunization Part 1 - Immunization Schedules (2017).

